# RAIRS Characterization of CO and O Coadsorption on Cu(111)

**DOI:** 10.1021/acs.jpcc.2c02541

**Published:** 2022-07-28

**Authors:** Diyu Zhang, Charlotte Jansen, Otto T. Berg, Joost M. Bakker, Jörg Meyer, Aart W. Kleyn, Ludo B. F. Juurlink

**Affiliations:** † Leiden Institute of Chemistry, Leiden University, P.O. Box 9502, 2300 RA Leiden, The Netherlands; ‡ Department of Chemistry and Biochemistry, Fresno State University, Fresno, California 93740, United States; ¶ Radboud University, Institute for Molecules and Materials, FELIX Laboratory, Toernooiveld 7, 6525 ED Nijmegen, The Netherlands

## Abstract

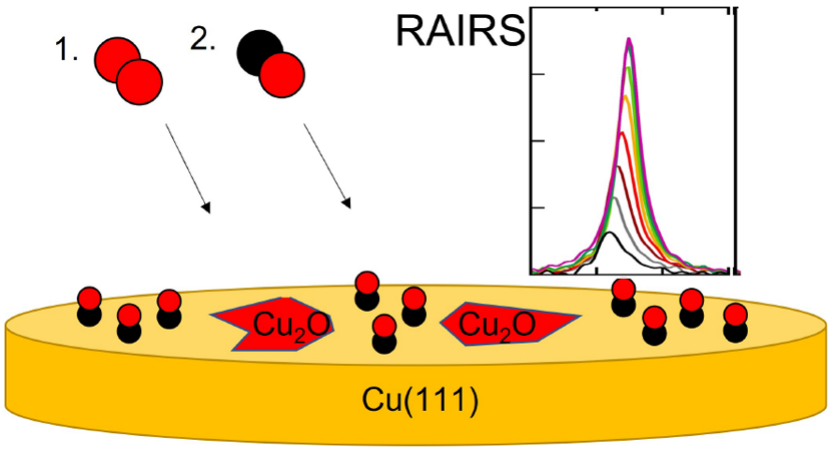

In a study preliminary to investigating CO_2_ dissociation, we report our results on oxygen and carbon monoxide coadsorption on Cu(111). We use reflection adsorption infrared spectroscopy and Auger electron spectroscopy to characterize and quantify adsorbed species. On clean Cu(111), the CO internal stretch mode appears initially at 2077 cm^–1^ for a surface temperature of ∼80 K. We accurately reproduce the previously determined redshift of the absorption band with increasing CO coverage. We subsequently oxidize the surface by exposure to O_2_ at 300 K to ensure O_2_ dissociation. The band’s frequency and line shape of subsequently adsorbed CO at ∼80 K are not affected. However, the maximum absorbance and integrated peak intensities drop with increasing O coverage. The data suggest that CO is not adsorbed near O, likely as a consequence of the mechanism of Cu(111) surface oxidation by O_2_ at 300 K. We discuss whether our RAIRS results may be used to quantify CO_2_ dissociation in the zero-coverage limit.

## Introduction

Methanol is an important industrial bulk chemical. It is used for the synthesis of, a.o., formaldehyde and acetic acid. It is also considered a potential energy carrier and may be used directly in methanol fuel cells to convert chemically stored energy into electricity. Industrial methanol synthesis utilizes copper-based catalysts and a mixture of CO_2_, CO, and H_2_. The process has been studied for several decades and—in light of world’s required CO_2_ emission mitigation—has recently inspired new discussions on the reaction mechanism and the role of various types of sites on the catalyst’s surface.^1−6^

Despite the history of studies on methanol formation from CO_2_, the dominant elementary reaction steps that constitute the chemical mechanism for this catalyzed process are not clearly determined. To unravel the reaction mechanism, experimental studies often use high-purity Cu single crystal surfaces for control over the structure and composition of the catalytic surface. Such surfaces are exposed to the reactants under conditions ranging from ultrahigh vacuum (UHV) to near-ambient pressure.^6−8^ Intermediates at the surface are often detected by techniques such as reflection adsorption infrared spectroscopy (RAIRS).^9^ Some experimental studies suggest that reaction is initiated by formation of an HCO_2_ intermediate from a direct, Eley–Rideal-like reaction of CO_2_ molecules with adsorbed H atoms,^10^ as was suggested by early theoretical studies.^11,12^ Recently, an important role of vibrational energy in the impinging CO_2_ for this direct reaction was suggested from a supersonic molecular beam study.^12^

The methods applied so far are, unfortunately, not sensitive to the sequence in which elementary steps occur. Under conditions where reaction is observed on single crystal surfaces, various IR peaks appear.^8^ These have been attributed to a number of species, e.g., surface-bound HCO_2_, HCO, OH, and CO. The appearance of these peaks does not provide evidence for the order in which elementary steps in the mechanism occur, as reaction time scales are much smaller than the acquisition time for these IR spectra. For example, formation of HCO_2_ may occur from oxidation of surface-bound HCO by an O atom, from internal rearrangement of surface bound OCOH, and from the hydrogenation of CO_2_ through direct insertion in the Cu–H bond. Although theoretical studies suggest the latter to occur and initiate formation of methanol, there is no prior evidence from experimental studies that would support a mechanism requiring insertion of CO_2_ in between the metal–H bond.

A complicating factor in experimental studies on reaction mechanisms involving CO_2_ is the very large discrepancy in the coverage-dependent sticking probability, *S*(θ), of CO_2_ and CO. While CO sticks to many well-defined surfaces of transition metals with a high (often near unity) sticking probability, CO_2_’s dissociative sticking probability, i.e., impingement leading directly to formation of adsorbed CO and O, is many orders of magnitude lower. In effect, apart from a single molecular beam study, Burghaus’s review on this topic reports no evidence of direct dissociative adsorption in gas-surface CO_2_ reaction dynamics studies.^13^ Usually, impingement energy-dependent sticking probabilities for similar dissociative events can be measured with supersonic molecular beam techniques at least down to 10^–6^.^14^ Consequently, CO contamination levels in a CO_2_ gas feed at the ppm level must already cause doubt on whether reaction products on a surface truly result from reaction of impinging CO_2_ as surface-bound contaminant CO is undoubtedly present. Considering that CO is also a dominant species in the residual gas in the often applied (ultra)high vacuum conditions for such studies, experiments that truly exclude interference by CO as a contaminant are very difficult. Use of isotopically labeled gases, e.g., ^13^CO_2_, is not helpful as these cannot be obtained with very high purity and may also contain ^13^CO. Especially at elevated nozzle temperatures in the expansion of gases from metallic nozzles to form supersonic molecular beams, unlikely chemical reactions take place,^15,16^ including ones that create CO from CO_2_ even if it was not present in a high-purity feed. In a recent study of this type, the presence of H_2_ converted over 80% of the CO_2_ feed into CO.^17^

A second complicating factor for experimental studies on elementary steps in methanol synthesis from CO_2_ results from the complex oxidation of Cu surfaces. Oxidation of Cu has also been studied for many decades, but only recent studies have definitively shown that oxidation is facilitated by defect sites, such as step edges.^18−22^ At low O_2_ exposures at room temperature no evidence of ordered structures on Cu(111) was found in a combined scanning tunneling microscopy (STM) and low energy electron diffraction (LEED) study.^19^ A study using a dome-shaped Cu crystal (*d*-Cu(111)-10° in the notation suggested by Auras and Juurlink^23^) showed that the rate of oxidation depends on the surface concentration of defect sites, but not on the type.^22^ With increasing oxygen surface concentration, structural changes occur^24^ and high (local) surface oxygen concentrations lead to Cu_2_O thin films formation.^25^ At exposures exceeding ∼3 × 10^3^ L (Langmuir, 1 × 10^–6^ Torr × s), oxygen atoms are also incorporated into the Cu lattice.^21^ Recently, a combined theoretical and experimental study suggested a critical role for such subsurface oxygen to reactivity of CO_2_ on Cu(111).^26^

In an attempt to develop a method that may undeniably quantify the reactivity of CO_2_ onto clean and H-containing Cu surfaces, we first report here on our RAIRS study of coadsorbed CO and O on Cu(111). Direct dissociation of CO_2_ is expected to generate O and CO that are, at least initially, coadsorbed at a small distance. A recent theoretical study on dissociation of CO_2_ on Cu(111) finds that the fragments end up in the top (CO) and 3-fold hollow (O) sites separated by 2/3√3 lattice spacings.^27^ For Ni(100)^28,29^ and multiple (100) structured metal surfaces,^30^ this is at most 1.5 lattice spacings. As the CO vibrational frequency is often dependent on its chemical surroundings,^31,32^ we hypothesize that RAIRS may distinguish CO randomly adsorbed to Cu(111) from a contamination source and CO_2_-generated CO by the respective absence and presence of the nearby adsorbed O atom. There is no *a priori* reason to assume that the coadsorbates will phase separate. Early ^12^CO/^13^CO isotopic dilution studies suggested that CO island formation at lower coverage does not occur on (oxidized) Cu(111),^32,33^ even though the CO diffusion barrier is experimentally determined^34^ and calculated^35^ to be rather low. Hence, we have no reason to expect that adsorbed CO will phase separate from O, leaving the CO frequency unaffected by coadsorbed O. Potential changes in the CO vibrational frequency, line shape, or intensity as a consequence of coadsorbed O may, therefore, uniquely identify CO_2_-generated CO and yield information on the details of the interaction between the adsorbates.^36^

The adsorption of CO to clean Cu(111) has been studied experimentally by RAIRS and other techniques in great detail over several decades.^33,37−48^ On clean Cu(111), the linear (on-top) C–O stretch frequency at 2076 cm^–1^ has been shown to present a modest, but characteristic, red shift with increasing coverage.^33,45,48−50^ A density functional theory (DFT) study suggested this mode to be susceptible to nearby adsorbed O atoms with a 65 cm^–1^ blue shift for the C–O stretch frequency from their original band at 2095 cm^–1^ for submonolayer coverages (θ_*O*_ = 1/4 ML, θ_*CO*_ = 1/9 ML) on Cu(111).^35^ The adsorption sites of CO and O in this study were also identical to those suggested by the previously referenced theoretical study of CO_2_ dissociation.^27^ This study of the influence on the stretch frequency also found a substantial shift of +34 cm^–1^ if oxygen atoms are incorporated in between the first and second Cu layers at the same surface concentrations. An earlier experimental study of CO adsorption to oxidized Cu(111) indicated that the single characteristic mode at approximately 2075 cm^–1^ for the clean surface was replaced by a weak doublet near 2100 and 2122 cm^–1^.^32^ It is difficult to compare the CO stretch frequencies, however, as the oxidation level of the Cu(111) surface in the experimental study was defined in terms of the measured surface potential, which we can not convert to an oxygen coverage or O_2_ dose. From our results, it will be clear that this latter experimental study likely dealt with a strongly oxidized surface and not with a surface that had submonolayer amounts of coadsorbed O and CO, as was the case in the theoretical study.

## Methods

In this study, we use Auger electron spectroscopy (AES) to quantify preadsorbed oxygen from molecular O_2_ dissociation and monitor the coverage-dependent characteristics of coadsorbed CO with RAIRS on Cu(111). The experiments were performed in a home-built ultrahigh vacuum (UHV) apparatus that has been described before.^51^ Its base pressure is below 2 × 10^–10^ mbar as measured by an uncalibrated hot cathode nude UHV ion gauge (Varian UHV-24) with a multigauge controller (Varian L8350-301). The apparatus is mainly used to perform Auger electron spectroscopy (ESA100, Staib Instruments) and reflection absorption infrared spectroscopy (Vertex70, Bruker) with an external liquid nitrogen cooled mercury cadmium telluride detector (LN-MCT Mid, Bruker). A residual gas analyzer (QMA200, Pfeiffer) may also be used to perform temperature-programmed desorption (TPD) spectrometry. TPD spectra suffer from strongly varying background signals, making quantitative treatment of the data less reliable. Otherwise, the system is equipped with a sputter gun (IS40 C1, Henniker Scientific) and various leak valves.

The sample used here is a Cu single crystal that was oriented and cut to the (111) plane within ±0.1°(Surface Preparation Laboratories, Zaandam, The Netherlands). The sample is laser-welded into a thin, U-shaped high purity Cu ring that allows for easy attachment to the bottom of a liquid nitrogen-cooled cryostat. The cryostat is suspended from an *x*,*y*,*z*,θ manipulator. The sample is heated by radiative heating and electron bombardment from an electrically isolated tungsten filament that is held approximately 1 mm behind the crystal. The crystal’s temperature is measured by a K-type thermocouple. It is laser-welded onto the crystal’s edge in between the legs of the U-shaped ring.

Before experiments, repetitive cleaning cycles remove contamination from the surface. Argon ion sputtering is performed at normal incidence (10 min, 500 V, ∼2 μA, at a surface temperature, *T*_surf_, of approximately 400 K). Sputtering is followed by annealing at 800 K (10 min). This procedure is repeated at least three times prior to every experiment. We regularly check for impurities by AES. Experiments are only performed if C, O, and S impurities are below our detection limit.

Dosing of gases is performed using standard leak valves. For dosing O_2_, the gas is introduced with the Cu(111) facing the leak valve at *T*_surf_ = 300 K, but there is no directed flow of O_2_ from the leak valve’s orifice. For dosing CO, a 6 mm diameter stainless steel tube is attached to another leak valve’s outlet to direct the flow of CO. It is aimed at the Cu(111) surface at normal incidence and a distance of approximately 30 mm during CO exposure. Dosing is done at *T*_surf_ = 80 K.

RAIRS spectra were recorded at 2 cm^–1^ resolution. The infrared light path is enclosed by a purge box which is continuously supplied with dry N_2_ gas. The purge box is isolated from the UHV chamber by CaF_2_ windows. IR light from the spectrometer is introduced at a grazing angle of ∼2.5°.

## Results

**Figure 1:**
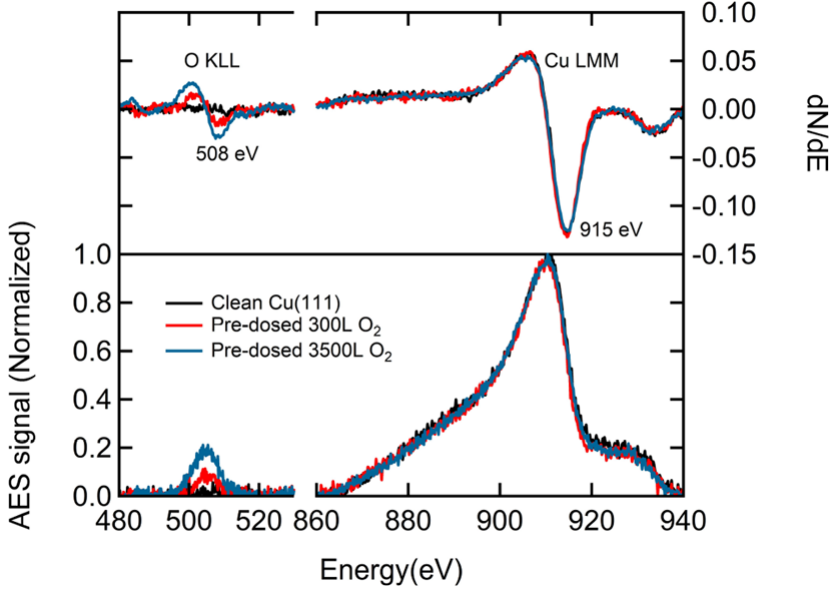
Normalized AES signals (lower pane) and differentiated spectra (upper pane) of the oxygen and Cu regions obtained after different exposures of Cu(111) to O_2_ at 300 K.

Figure 1 shows representative AES spectra for the O and Cu regions taken after various exposures of the clean Cu(111) surface to O_2_ at *T*_surf_ = 300 K. The bottom part of the figure shows background-corrected raw spectra. In the upper panel, we report the differentiated scans. All AES data were normalized to the Cu LMM peak at 910 eV. Minor variations in conditions and settings from experiment to experiment induce significant changes in the intensity and shape of the background signal of AES spectra and require this normalization. After dosing of O_2_ at *T*_surf_ = 300 K, which is known to result in dissociative adsorption (see, e.g., refs (52−54)), we observe the KLL peak of oxygen. It appears between 490 and 520 eV. The oxygen KLL peak intensity clearly increases with O_2_ exposure.

**Figure 2:**
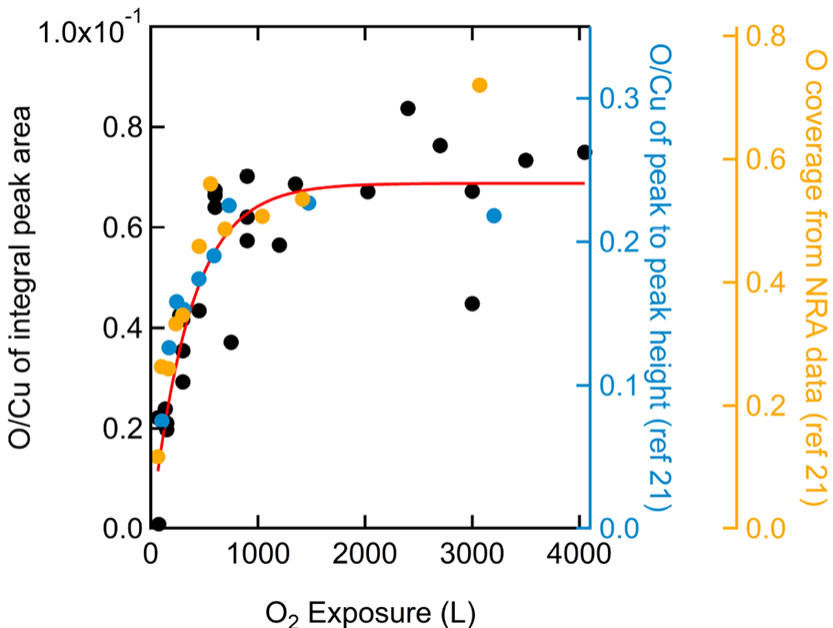
Oxygen-to-copper AES integrated (left) and derivative (first right) signal ratios and O coverage (second right) as a function of O_2_ exposure. Our data are shown as black symbols with a best fit using an appropriate functional form for precursor-mediated adsorption shown in red. Data extracted from ref (21) are plotted as blue and orange symbols. Vertical axes are color-coded to match the data. Significant scatter in our data is thought to result mostly from instability of the background signal around O emission and the required background fitting that precedes peak integration.

We quantify the oxygen coverage by integrating our AES spectra. The oxygen KLL peak is integrated from 490 to 520 eV. We use the integrated Cu LMM peak from 860 to 940 eV as an internal standard. The ratios of the oxygen-to-copper integrated areas are plotted as a function of O_2_ exposure in Figure 2 (black symbols). Even though there is significant scatter, the data suggest that the absorbed oxygen increases with O_2_ dose until saturating at approximately 1–1.5 × 10^3^ Langmuir (L).

The conversion of the AES O-to-Cu intensity ratio to an absolute oxygen coverage is not unambiguous. We use a primary energy of 3 keV when collecting AES spectra. Hence, we may expect a contribution to AES signals from the selvedge. The contribution to the Cu signal from the topmost layers should drop modestly as an oxygen overlayer is created. Thus, normalizing all spectra initially to the intensity of the main Cu peak, as if the Cu contribution is not changing, artificially enlarges the AES signal for O. As Cu(111) surface oxidation is not expected to be self-limiting, the measured oxygen signal may at some point also start reflecting O atoms being incorporated into the selvedge, not only from those adsorbed on top of the surface.^21^ Incorporation into the selvedge also enlarges the O signal, even though the actual surface concentration is not increasing anymore. The gradual flattening of the AES intensity ratio in Figure 2 beyond 1000 L O_2_ suggests that the rate of continued oxidation is much smaller, however, than the initial rate of oxygen adsorption to the clean Cu(111) surface. For lack of better criteria and other types of data from our own laboratory that may help in a more accurate conversion, we assume that at the highest exposures of 4000 L the surface has oxidized forming a skin layer with an atomic O-to-Cu ratio of 0.5,^21^ i.e., Cu_2_O. The second right axis in Figure 2 reflects the conversion and also serves as a reference for nuclear reaction analysis (NRA) data on oxidation of Cu(111) as collected by Jensen et al.^21^ Their AES data are also shown in blue for the first right axis. They used a peak-to-peak height analysis that yields a different maximum value for the ratio (∼0.24) than our ratio of integrated peaks (∼0.07) but also shows a limiting value for exposures beyond 10^3^ L, whereas NRA data suggests subsequent incorporation into the selvedge. The red line in Figure 2 is a best fit to the data using the appropriate functional form for indirect (i.e., precursor-mediated) adsorption leading to dissociation and filling of surface sites. Its shape is very similar to the one used in a previous study that also quantified O adsorption to Cu(111) by AES and suggested saturation above a 1000 L dose.^20^

**Figure 3:**
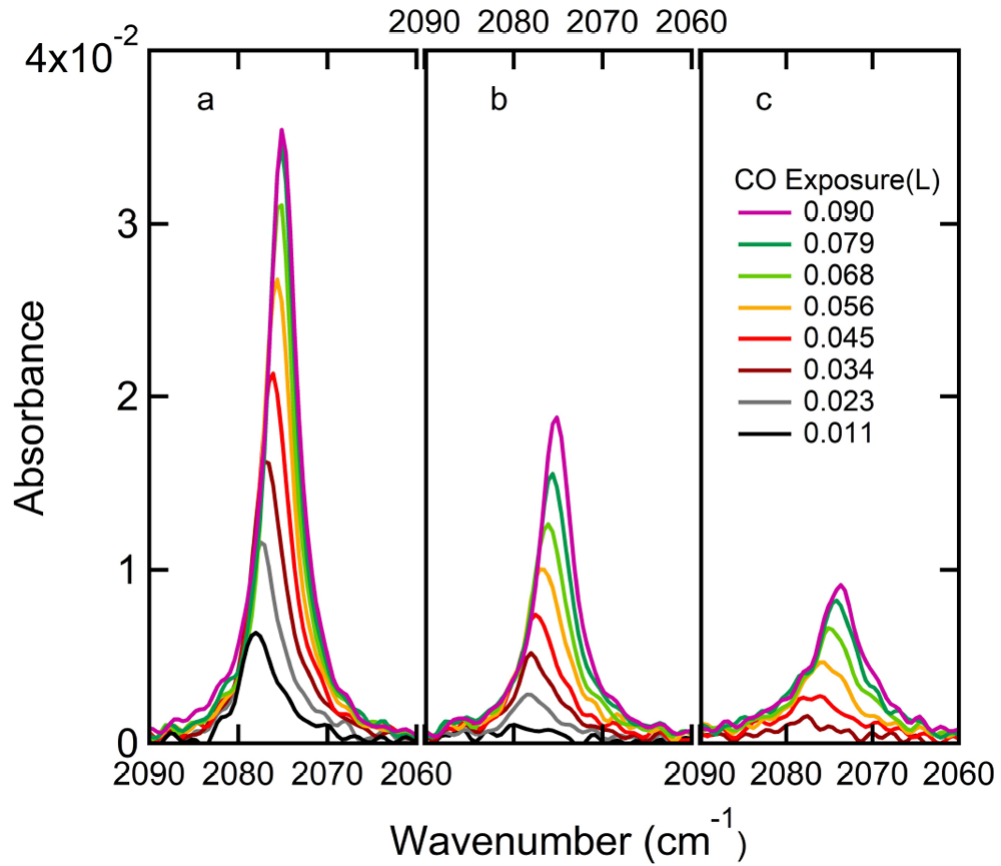
RAIRS spectra of sequentially dosed CO on (a) clean Cu(111), (b) 150L O_2_ pre-exposed Cu(111), i.e., ∼0.17 ML O_ads_, (c) 225L O_2_ pre-exposed Cu(111), i.e., ∼0.24 ML O_ads_. The CO dose is indicated in the legend.

We subsequently studied the adsorption of CO onto clean and O_2_ pre-exposed Cu(111) using RAIRS. Figure 3 shows three representative sets of IR spectra for increasing CO exposures at a surface temperature of 80 K. The left panel shows spectra for CO adsorbed to the clean Cu(111) surface. In the middle panel, an initially cleaned Cu(111) surface was first exposed to 150 L of O_2_ at 300 K. This corresponds to an estimated O-coverage of 0.17 ML. For the right panel, 225 L of O_2_ was dosed. This corresponds to an estimated O coverage of 0.24 ML. The vibration of the chemisorbed CO internal stretch mode, centered between 2080 and 2070 cm^–1^, increases in intensity and shifts to lower frequencies with increasing CO dosage up to 0.090 L. The same phenomenon has been observed by previous RAIRS detailed studies of CO/Cu(111).^45,48^ In our work, the physisorbed CO stretch vibration near 2140 cm^–1^ observed previously at surface temperatures of 7 K^45^ and 25 K^48^ is not observed, most likely because of our higher surface temperature. At 77 K, Hayden et al. also did not find this characteristic frequency.^45^ We also find no clear evidence of the double absorption previously reported by Hollins and co-workers above 2100 cm^–1^.^33^ Figure S1 in the Supporting Information shows typical spectra with broader frequency ranges. Increasing the pre-exposure to O_2_ lowers the rate at which the IR absorption increases with CO coverage. The IR absorption at 0.09 L CO exposures (i.e., the largest shown in Figure 3) has dropped nearly 4-fold when comparing the pre-exposed surface at 225 L O_2_ to the clean Cu(111) surface.

**Figure 4:**
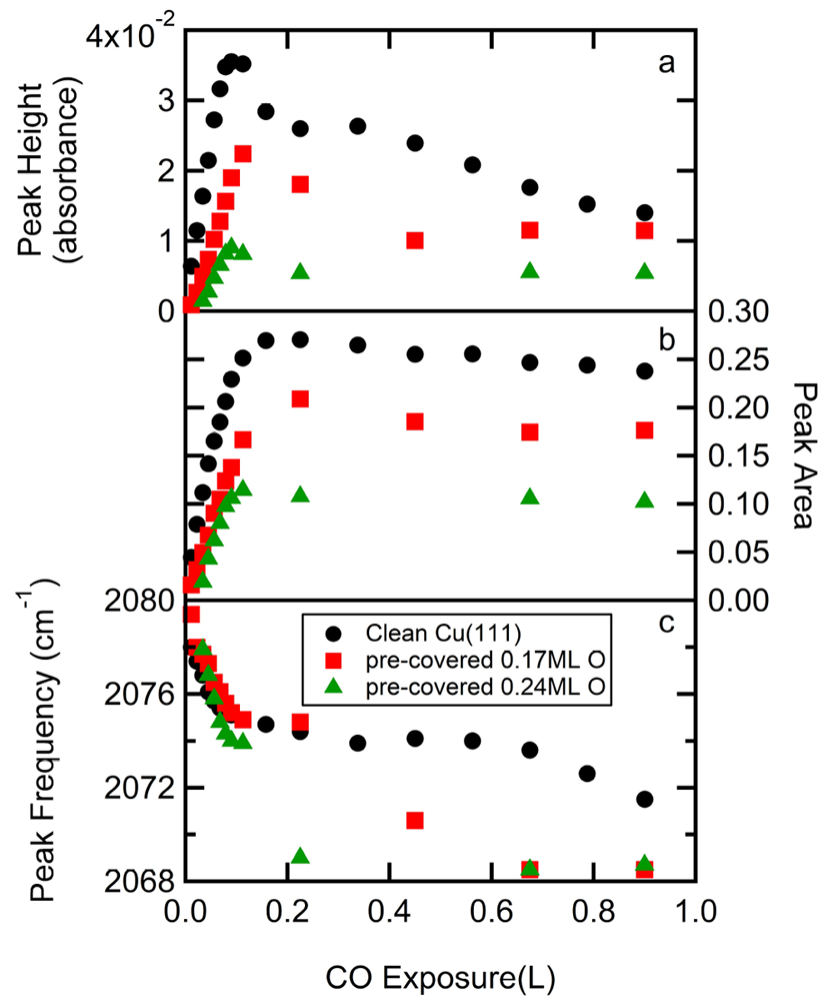
Development of three characteristic values resulting from CO IR adsorption profile fits as a function of CO dose for three representative data sets of predosed oxygen coverages: (a) peak height, (b) peak area, and (c) peak frequency. The parameters result from pseudo-Voigt profile fits to IR spectra such as shown in Figure 3. The resulting O_ads_ coverages estimated from O_2_ predoses for three data sets (black (circles), red (squares), green (triangles)) are specified in the legend.

To obtain accurate parameters reflecting the IR absorption by CO, we fit the absorption spectra using a modified asymmetric pseudo-Voigt profile.^55^ Figures S2 and S3 in the Supporting Information show typical fits and reflect the quality of the procedure. Figure 4 shows results extracted from the obtained fitting parameters. These are plotted as a function of CO exposure for the same oxygen predose conditions used in Figure 3. The absorbance at the peak frequency is shown in the top panel, Figure 4a. From the fitted function, we calculate the integrated band intensity (”peak area”). It is reported in the center panel, Figure 4b. Finally, the same fits also provide the frequency at maximum absorbance (ν_*p*_ or peak frequency). It appears in the bottom panel, Figure 4c. The data are color-coded. Black (circles) represents the clean Cu(111) surface. Red (squares) and green (triangles) represent pre-exposures to 150 and 225 L O_2_, respectively. Note that the CO dose range in Figure 4 is increased 10-fold in comparison to the range of Figure 3.

We identify two regimes for the attained IR characteristic dependencies on the CO dose and the precoverage of O_2_. First, over the range of 0 to ∼0.1 L of CO exposure, ν_*p*_ redshifts with CO dose. The shift is hardly (if at all) affected by preadsorption of oxygen. The shift is linear with CO dose in all cases from ∼2078 to 2074 cm^–1^. At the same time, the peak intensity (in terms of the peak height and peak area) scales linearly with CO exposure. The maximum peak height and peak area, observed near 0.1 L CO for all O_2_ pre-exposures, drop with the O precoverage.

In the second regime, i.e., beyond ∼0.1 L CO exposure, ν_p_ continues to shift downward toward 2069 cm^–1^, but the rate is now clearly dependent on the O_*ads*_ coverage. Overall, the IR absorption feature broadens in this regime and the peak height clearly drops. For the non-oxidized Cu(111) surface, the peak height drops by a factor of 3 over this range. The peak area in the center panel remains, in contrast, comparatively constant between 0.1 and 1 L CO exposures. In all cases, it drops by approximately 10% over this range.

**Figure 5:**
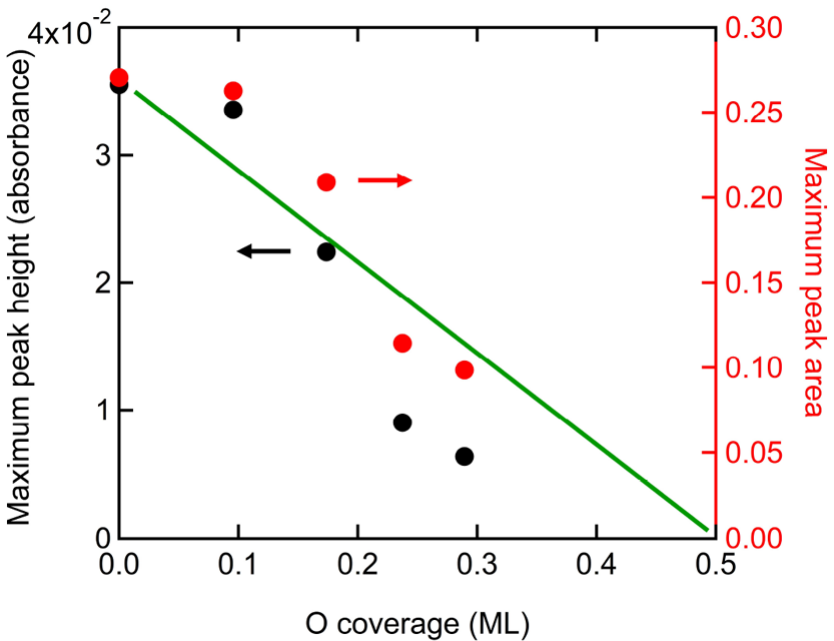
Oxygen coverage dependence of the CO maximum peak height (left axis) and maximum peak area (right axis) for the stretch IR absorption of linearly bound CO on Cu(111) at 80 K. The green line is a linear function connecting data for the clean Cu(111) surface, i.e., zero oxygen coverage, to a value of 0 peak height and peak area for 0.5 ML O/Cu(111).

To check whether characteristic values that we may extract from Figure 4 are linearly dependent on O precoverage, we plot in Figure 5 the dependence of two such values. First, the maximum CO absorbance for the various O precoverages is extracted from Figure 4a and plotted in Figure 5 against the left axis (black circles). We do the same for the maximum CO peak area and plot it against the right axis (red circles). The data in Figure 5 also show the same for two additional initial oxygen precoverages (i.e., 0.096 and 0.29 ML O) that were omitted from Figure 4 for reason for of clarity. The vertical axes in Figure 5 have been adjusted to have the first data point from both parameters overlap. The trend for both indicators is roughly the same but clearly deviates from a linear dependence on the O precoverage. The green linear function is added for reference only and connects the initial data for the clean Cu(111) surface with zero peak height and peak area for the fully oxidized Cu(111) surface.

## Discussion

Auger signals with our equipment are quite small and seem sensitive to minor changes in experimental settings. The AES intensity ratio of O and Cu in Figure 2 is also rather small, approaching only 0.07 for the seemingly O-saturated surface. The comparison to previous data from Jensen et al.,^21^ shown in the same figure for reference, supports the conversion of the O_2_ dose to an actual surface oxygen coverage, however. In their experiments, NRA was combined with AES to determine the relationship between oxygen exposure, the AES signals for O and Cu, and the absolute O coverage on Cu(111). They concluded that the (111) surface oxidizes to the coverage of 0.5 ML O/Cu during exposure up to ∼10^3^ L as AES and NRA data tracked each other. Beyond this dose, O is much more slowly incorporated into the selvedge as shown by increasing NRA signals for oxygen while AES signals stop increasing. Our O_2_ exposure-dependent AES ratios in Figure 2 agree quite well with those results despite significant scatter in our data. Hence, we feel confident that we can use the reliably determined O_2_ exposure up to ≤1500 L to quantify the obtained O-coverage through the fit to our data.

The coverage dependencies in our RAIR spectra of CO for the clean Cu(111) surface are also in very good agreement with the most detailed previous RAIRS study which used a nearly identical surface temperature.^45^ There, an initial linear increase in the integrated intensity and red shift of the center frequency from 2078 to 2074 cm^–1^ were reported. The redshift of the peak frequency was previously assigned to a chemical nondipole coupling effect.^56^ These linear changes occur over a slightly broader CO dose range (up to approximately 0.2 L) than in our results (up to approximately 0.1 L). The difference is likely due to variations in pressure gauge sensitivities and/or the geometry used in dosing CO onto the cold surface. The latter is highly directed in our case and the local flux at the surface likely exceeds the corresponding pressure detected by the pressure gauge elsewhere in the UHV chamber.

Beyond the initial dose regime with linear changes in spectral characteristics, the center frequency was reported to stabilize while the peak area modestly dropped up to a 1 L CO dose.^45^ This is also identical to our findings. In the same regime of CO dose, low energy electron diffraction (LEED) patterns implied initial short-range ordering of adsorbed CO into a (√3 × √3)*R*30° structure with CO bound linearly to Cu atoms, but only after the surface was annealed from the adsorption temperature of 77 to 100 K. It implies a disordered structure at 77 K with a coverage increasingly approaching 0.33 ML CO but never settling into the well-ordered structure. Only beyond a 1 L dose was a 1.4 × 1.4 hexagonal overlayer structure containing bridge-bound CO (1835 and 1814 cm^–1^) found without annealing. As we do not anneal our surface and dosed CO at 80 K, we may safely assume that in the same dosing regime, CO also adsorbs in our studies in a disordered fashion with the surface concentrations approaching 0.33 ML for a 1 L dose.

Our data for O-precovered Cu(111) show that the nature of CO adsorption to Cu(111) is not significantly affected by the presence of oxygen. The linearly adsorbed CO stretch vibration appears at the same initial frequency of ∼2078 cm^–1^ and shifts linearly to ∼2074 cm^–1^ when the CO exposure increases from 0.011 to 0.1 L. This occurs for all oxygen precoverages of which three sets are shown in Figure 4c. However, the peak height and peak area increase less rapidly and show a clear dependence on the amount of precovered oxygen. This can be deduced from the slopes in the data in Figures 4a,b. The increase stops at lower values, and the lowered maxima were shown in Figure 5. The combination of an unchanged (shift in) peak frequency with dropping (maximum) absorbances suggests that the adsorbed CO is only bound to Cu(111) areas that are unperturbed by the prior oxygen adsorption. With increasing O_2_ exposure, less Cu(111) remains unaffected and less CO adsorbs, but in an identical manner as to pristine Cu(111). Our results are, therefore, most logically explained in terms of separated patches or phases of oxidized Cu(111) and pristine Cu(111) with CO only binding to the latter. We expect that the patches of CO/Cu(111) are without clear order. The lack of LEED optics on our UHV system unfortunately makes it impossible to verify this. The lower rate of adsorption of CO onto the partially oxidized surface, as reflected by slower increase in peak height and peak area, suggests that the sticking probability of CO impinging onto Cu_2_O(-like) patches is smaller than that of Cu(111).

Previous STM studies provide support for our interpretation. Dissociative adsorption on Cu(111) and vicinals occurs preferentially on step edges and kink defects.^19,22,57^ Small defect islands rapidly oxidize to form triangular islands that grow with O_2_ exposure. A very similar oxidation mechanism was shown to occur on silver using a *c*-Ag(1 1̅1)[110]*R*31° crystal.^58,59^ Our current RAIRS results indicate that the remaining nonoxidized Cu(111) patches after partial oxidation of the surface are electronically unperturbed, because the center of the CO band frequency does not change. We only find that the clean Cu(111) area decreases with increasing O_2_ exposure. Hence, a patch-wise oxidation mechanism with CO adsorbing only the unoxidized Cu(111) at 80 K is consistent with our—in effect—titration of the remaining pristine Cu(111) area.

The interpretation that CO only binds to the pristine Cu(111) may seem remarkable as CO has been found to adsorb under UHV conditions to the Cu_2_O(100) surface of a Cu_2_O single crystal.^60^ CO was even found to have a significantly higher desorption temperature and binding energy (69 kJ/mol) than on Cu(111) (58 kJ/mol).^33^ Moreover, CO can be oxidized on Cu_2_O particles with a recent study employing cubic particles of various size to identify the oxidation sites.^61^ This seeming contradiction is easily explained, however. The oxidized Cu(111) patches do not adsorb CO in our experiments as they are structurally different from the surfaces present on pristine Cu_2_O particles. This may also be expected from the results of another recent oxidation study on the density and orientation of Cu_2_O surfaces and particles grown under much higher O_2_ pressures from Cu(111), Cu(110), and Cu(100).^62^ Oxide nucleation was found to depend on the type of vicinal surface. Hence, although we designate the oxidized Cu(111) surface as Cu_2_O(-like), this merely reflects the elemental ratio as determined previously for our conditions.^21^

For a system containing patches of Cu(111) and some Cu_2_O-like surfaces, one may expect a strictly linear dependence for the CO maximum peak height and maximum peak area on oxidized area or oxygen coverage. Such a trend is suggested in Figure 5 by the linear function. We find, however, that both the maximum peak area and peak height are somewhat higher below Θ_O_ = 0.2 ML. Beyond Θ_O_ = 0.2 ML both values are lower. We speculate that these two IR characteristics reflect heterogeneity in CO island sizes that result from heterogeneity in Cu_2_O patch sizes. The data may, therefore, not strictly follow a linear dependence, although they are apparently not far from it. The difference in the O-coverage dependence for the maximum peak height and peak area in Figure 5, i.e., the data not overlapping, reflects minor variations in peak shapes.

An interesting aspect to our data is the more rapid drop in frequency beyond 0.1 L CO exposure for preoxidized surfaces than the clean Cu(111) surface. The frequency drops ultimately to the value of 2068 cm^–1^ in Figure 4c. As the absorption profile also broadens, we assume that the drop from 2074 to 2068 cm^–1^ is mostly due to increasing disorder in the CO/Cu(111) adlayer. The low rate of this occurring for the non-oxidized Cu(111) surface (it takes more than an extra 1 L of CO exposure) suggests that this results from a low sticking probability of CO onto/into CO-precovered patches. The fact that the drop to the ultimate frequency is attained faster for preoxidized surface suggests that these oxidized areas help in attaining the ultimate local CO coverage and peak frequency on the CO-binding nonoxidized Cu(111) patches. Hence, although they do not seem to bind CO stably at 80 K themselves, the oxidized copper does capture CO and allows it to diffuse to the CO/Cu(111) patches.

Finally, we consider the difference between our current experiment and an experiment actually starting with CO_2_ dissociation on Cu(111). Owing to the extreme low dissociation probability of CO_2_, we created the coadsorbed system by sequential dissociative adsorption of O_2_ and molecular adsorption of CO here. These methods are clearly not identical and may *a priori* lead to different distributions or ordering of CO and O on the surface. As the procedure used in the current study appears to create a coadsorbed system with CO on pristine Cu(111) patches and oxygen atoms accumulating in Cu_2_O patches, we cannot conclude whether it is possible to differentiate between CO_2_-generated CO and contaminant CO on the basis of CO’s absorption frequency. We surely do not see the large shifts in this frequency as suggested by theory.^35^ In an experiment where CO_2_ is the source of CO and O, the CO IR absorption characteristics may still differ from what we find here and agree with the predictions for the DFT calculations. Also, the rather strong dependencies of the maximum CO peak height and area on oxygen coverage, as reported in Figure 5, may be used in titration experiments. With O atoms resulting from CO_2_ dissociation and these blocking CO adsorption, the O-coverage may be probed by subsequent incremental dosing of CO and recording the maximum peak height and area of the IR absorbance. Hence, in the absence of other techniques or surface science tricks, RAIRS of (post-adsorbed) CO may still provide a means to unambiguously quantify direct CO_2_ dissociation.

## Conclusion

Our study of CO and O coadsorption was intended to determine whether we could distinguish CO generated by CO_2_ dissociation from CO randomly adsorbed from the UHV residual gas or other contamination sources. The concern was motivated by the wish to study CO_2_ direct dissociation in the low-coverage limit and the vastly varying sticking probabilities of CO_2_ and unavoidable CO contamination. The results of the described experiments that use O_2_ dissociation to introduce O atoms prior to adsorbing CO on Cu(111) do not provide conclusive evidence. The most characteristic signature within RAIRS of CO, i.e. the IR absorption frequency, is indistinguishable. We ascribe it to the oxidation mechanism of Cu(111) by O_2_ and an apparent inability of Cu_2_O patches to chemically bind CO. On the other hand, we do find strong dependencies in the maximum absorbance and peak area that may be used to quantify the amount of O on a Cu(111) surface using CO titration.

## Supporting Information Available

Additional RAIR spectra over a broad frequency range and typical fits of the modified pseudo-Voight profiles to CO absorption peaks (PDF)
