# Supplementary material for: RAIRS Characterization of CO and O Coadsorption on Cu(111)
Source: J Phys Chem C Nanomater Interfaces. 2022 Jul 28;126(31):13114–21. doi: 10.1021/acs.jpcc.2c02541 (PMC9376953; doi:10.1021/acs.jpcc.2c02541)
Supplement: Supplementary file 1 — jp2c02541_si_001.pdf [file jp2c02541_si_001.pdf]

RAIRS Characterization of CO and O Coadsorption on Cu(111)

Diyu Zhang,† Charlotte Jansen,† Otto T. Berg,‡ Joost M. Bakker,¶ Jörg Meyer,† Aart W. Kleyn,† and Ludo B. F. Juurlink*,†

†*Leiden Institute of Chemistry, Leiden University, PO Box 9502, 2300 RA Leiden, The Netherlands*

‡*Department of Chemistry and Biochemistry, Fresno State University, Fresno, CA 93740, USA*

¶*Radboud University, Institute for Molecules and Materials, FELIX Laboratory, Toernooiveld 7, 6525 ED Nijmegen, The Netherlands*

E-mail: l.juurlink@chem.leidenuniv.nl

Phone: (+31) 71 527 4221

Supporting Information Available

Figure S1 shows three RAIR spectra over a broad frequency range that would include physisorbed CO and bridge-bound CO. We find no evidence of other species than linearly (on top) bound CO.

Figures S2 and S3 show two typical fits of the modified pseudo Voight profiles to CO absorption peaks. Figure S2 shows the RAIR spectrum and the fit to CO bound to clean Cu(111) after a dose of 0.09 L CO resulting from exposure to 5 × 10^{-10} mbar CO. Note that our dosing configuration ensures aiming CO at the T_surf = 90 K surface and that the pressure increase likely does not fully reflect the local CO flux. Figure S3 shows the same

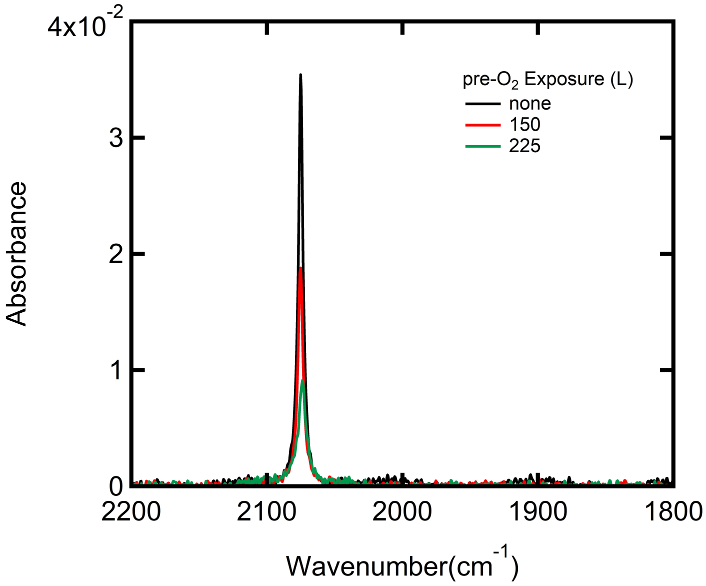

Figure S1: RAIR spectraof 0.09 L CO on Cu(111) with various pre-doses of O_2 amount.

for a Cu(111) surface pre-exposed to 150 L O_2, corresponding to an estimated O-coverage of 0.17 ML.

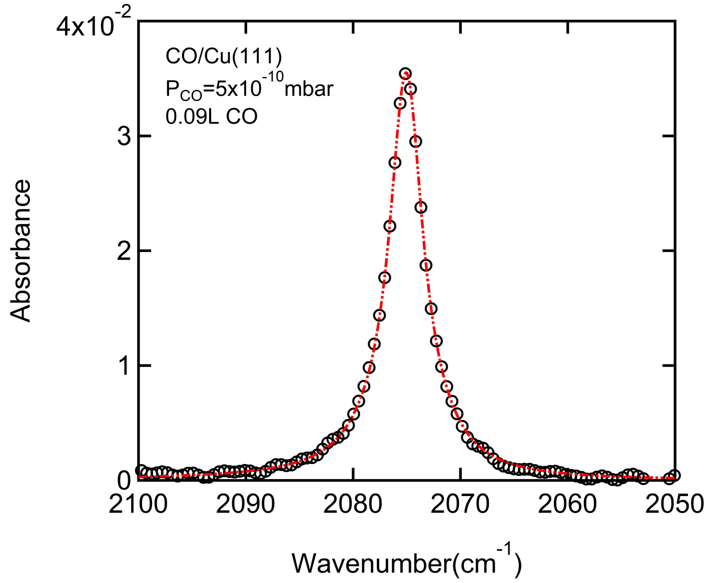

Figure S2: Exemplary spectrum and fit of absorbed CO (open circles) on clean Cu(111) with the asymmetric pseudo-Voigt model (red dotted line).

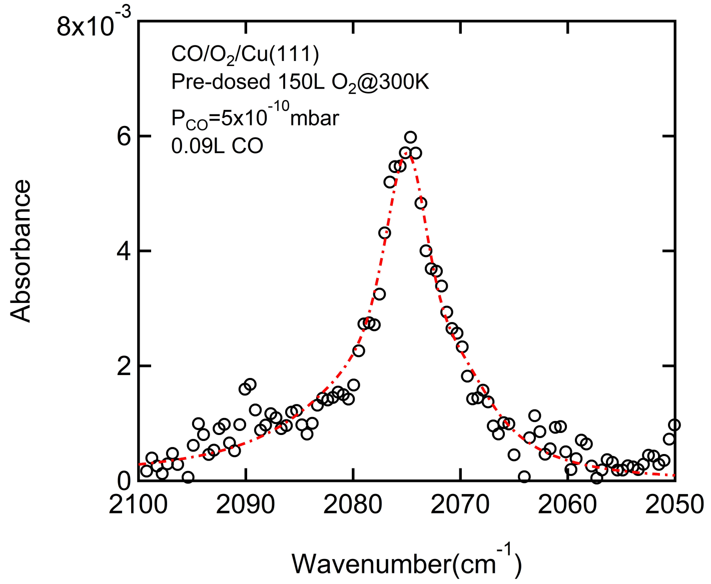

Figure S3: Exemplary spectrum and fit of absorbed CO (open circles) on O-precovered Cu(111) at 0.17 ML O with the asymmetric pseudo-Voigt model (red dotted line).
